# Health Behavior, Health-Related Quality of Life, and Mental Health Among Canadian Children: A Population-Based Cohort Study

**DOI:** 10.3389/fnut.2021.638259

**Published:** 2021-03-11

**Authors:** Xiuyun Wu, Paul J. Veugelers, Arto Ohinmaa

**Affiliations:** School of Public Health, University of Alberta, Edmonton, AB, Canada

**Keywords:** health behavior, diet quality, physical activity, sedentary behavior, health-related quality of life, internalizing disorder, attention-deficit and hyperactivity disorder, children

## Abstract

**Objective:** Studies that have reported the associations of diet quality, physical activity (PA), sedentary behavior (SB), and health-related quality of life (HRQoL) with mental health among children and adolescents are predominantly cross-sectional in design. Very few studies have examined the longitudinal relationship of mental health with health behavior and HRQoL among children. This study aimed to investigate the associations of diet quality, PA, SB, and HRQoL among children with mental health disorders throughout childhood.

**Methods:** We linked data from grade five students aged primarily 10 and 11 years who participated in the Raising Healthy Eating and Active Living (REAL) Kids Alberta survey in 2012 in the Canadian province of Alberta with their administrative health care data from birth to 2012. Mental health outcomes included internalizing disorder and attention deficit and hyperactivity disorder (ADHD) defined by the International Classification of Diseases, Ninth Revision, Clinical Modification (ICD-9-CM) or Tenth Revision, Canadian version (ICD-10-CA). The HRQoL was measured by the EQ-5D-Y, a five-dimensional descriptive system for children and youth. We applied negative binomial regressions to examine the associations between health behaviors, HRQoL, and mental health.

**Results:** Of the 1,352 participating students, 12.31 and 8.32% had a diagnosis of internalizing disorders and ADHDs, respectively, during childhood from birth to the ages of 10–11 years. Students in the highest tertile for diet quality, relative to the lowest tertile, were 56% less likely to have diagnoses of internalizing disorders (incidence rate ratio, IRR = 0.44, 95% CI = 0.23–0.85). Students engaged in less PA (*vs*. more PA) were more likely to be diagnosed for internalizing disorders (IRR = 1.98, 95% CI = 1.19–3.30). Poorer diet quality, low PA, excessive use of computers/video games, and watching TV were significantly associated with more diagnoses of ADHDs. Children who experienced some or a lot of problems in “feeling worried, sad, or unhappy” and “having pain or discomfort” were more likely to receive diagnoses of internalizing disorders and ADHDs, respectively.

**Conclusions:** These observed associations suggest that health promotion programs targeting promoting diet quality, PA, and HRQoL and reducing SB among children may contribute to improving mental health.

## Introduction

Childhood and adolescent mental health disorders are a significant global public health issue and constitute a considerable burden for public health and medical care systems (1). Approximately half of all lifetime mental health disorders begin in early childhood and adolescence before the age of 14 years (2). Poor mental health in children and adolescents is associated with lower health-related quality of life (3, 4), poor educational achievements (5), and increased risk of engaging in risky behaviors like substance use, self-harm, suicide attempts, and suicide (6). The mental health problems in childhood and adolescence often remain stable and persist into adulthood (7–9), and thus exacerbating the global burden of diseases with substantial disability-adjusted life years lost (10). Therefore, identification of modifiable risk factors for poor mental health in childhood is a high priority in order to develop health promotion initiatives among children.

Previous studies have documented the associations of health-related behaviors, including physical activity (PA) (11, 12), diet quality (13, 14), and sedentary behavior (SB) (15, 16), with mental health among children and adolescents. The evidence-based findings suggest that low levels of PA and high SB are associated with poor mental health, including depression, anxiety, emotional disorders, and other internalizing problems (11, 12, 16), and poor diet quality is associated with decreased mental health (13, 14) among children, and adolescents. However, the existing findings are predominantly based on cross-sectional investigations, and very few longitudinal studies have examined the importance of these health-related behaviors for mental health among children and adolescents (11, 13, 16). The health benefits of PA for mental health have been more described by researchers than the health benefits of SB and diets for mental health among children and youth (11–13, 16). Less research has been conducted to examine the effects of diet quality and SB on mental health among children and adolescents (11, 13). The existing findings for the relationship of SB and diet quality with mental health problems among children and youth appear considerably variable. Some studies found a significant association between SB or diet quality and mental health (17, 18), and others did not observe an adverse effect of elevated SB or poor diet quality on mental health (19, 20). Moreover, studies that investigated the importance of health behaviors for mental health in young people have mostly been carried out among adolescents, and considerably less research has been conducted among children and preadolescents (11, 13). Particularly, of the studies that have used a longitudinal design to examine the relationship of diet quality with mental health among children and adolescents, the majority of the studies had a short follow-up period (e.g., < 5 years) (17, 21) and have investigated the influence of diet quality on mental health among adolescents rather than children (13, 14). As healthy behaviors, like active lifestyles, healthy diet habits, and low SB are often established during childhood and persist into later in life (22, 23), it is essential to examine the relationships of PA, SB, and diet quality with mental health disorders among children to inform health intervention initiatives in childhood in order to enhance their mental health. In addition, most of the previous studies have examined mental health outcomes by utilizing self-reported measures of mental health indicators. Very few studies have used physician diagnosis of a mental health disorder indicating a clinically significant mental health problem (e.g., mental illness) (15).

The associations of mental health problems with health-related quality of life (HRQoL) have been mostly examined among adults (24) or among children and adolescents with chronic diseases (e.g., type 1 diabetes) or are overweight (25, 26). Very few population-based studies have analyzed the relationship between HRQoL and mental health among children and adolescents (3, 4, 27), and studies based on longitudinal data are scarce. HRQoL is a multidimensional construct that comprises the physical, psychological, and social functioning of an individual (28). It is important to examine which specific dimension or subscale of HRQoL (e.g., physical or psychosocial functioning) is related to mental health problems and in what manner (e.g., negative or positive association and strength of the association). More population-based studies among children are needed to better understand the relationships of mental health and HRQoL with multiple health behaviors and socioeconomic confounders being considered.

The purpose of this study was to examine the associations between diet quality, PA, SB, and HRQoL in children aged primarily 10–11 years and mental health disorders throughout childhood.

## Methods

### The Survey

The 2012 Raising Healthy Eating and Active Living Kids in Alberta (REAL Kids Alberta) survey was a population-based survey among grade five students aged primarily 10 and 11 years and their parents in the province of Alberta, Canada (29). The 2012 REAL Kids Alberta survey aimed to evaluate a comprehensive initiative by Alberta Health and Wellness to promote healthy behaviors and health among school children. The survey employed a one-stage stratified random sampling design with a sampling frame that included all elementary schools in the province. Schools were stratified according to residential regions (metropolitan, city, or rural town) to ensure proportional representation of schools in each geographic region (29). Of the 170 selected schools, 143 (84%) agreed to participate. A total of 2,673 students from the participating schools had home surveys returned, among which 2,427 (91%) provided parental consent for their child's participation, and 2,308 (95%) of these students completed the survey.

The REAL Kids Alberta survey included a home survey completed by parents and a student survey that was completed by students in the schools. The survey was administered to students during classroom time by trained assistants who also measured the standing height and body weight of the students. The student survey included the validated Harvard Youth/Adolescent Food Frequency Questionnaire (YAQ) (30, 31) adapted version for Canadian children and youth, questions on physical activities, sedentary behaviors (watching TV and playing computers or video games), and the EQ-5D-Y descriptive system (32). The home survey collected information on children's socio-demographic characteristics, including gender, place of residency, household income, and highest level of parental education. Parents were also asked to provide Alberta health insurance number for their child and consent for their child's survey information to be linked with the child's administrative health care records.

### The Administrative Health Data

The administrative health care data in Alberta included the Canadian Institute for Health Information Discharge Abstract Database (DAD), the physician services and claims database, and the ambulatory care database. The physician claims database includes physicians' services and billing information for these services. The DAD contains comprehensive administrative records for each inpatient admission to a hospital facility in Alberta. The Alberta ambulatory care database includes emergency department visits and outpatient day procedures. All of these databases contain individual patient-level information including patient demographic characteristics, diagnoses of diseases and procedures received, and services and treatments received. The health care data of the children used in this study were from 2000 (child's birth) to the end of March 2012 (before the survey began in April 2012).

Of the 2,308 students who completed the REAL Kids Alberta survey, 1,352 (59%) students with valid health care card numbers provided by their parents were successfully linked with the disease diagnosis records in the administrative health data.

### Mental Health Disorder Outcomes

The outcomes included the total number of physician visits, emergency department visits, and hospitalizations for internalizing disorders and attention deficit and hyperactivity disorders (ADHDs) throughout childhood. Mental health disorder was defined as a mental disorder by a physician's diagnosis using the International Classification of Diseases, Ninth Revision, Clinical Modification (ICD-9-CM) or Tenth Revision, Canadian version (ICD-10-CA). The ICD-9-CM and ICD-10-CA codes for the mental health internalizing disorders included “296, 296.2–296.3, 296.6–296.9, 300, 308, 309, 311, 313, F32–F34, F38–F43, F48, F92, and F93” (20, 21). These codes cover the diagnostic groups of depressive episode and recurrent depressive disorders, mood disorders (excluding bipolar), neurotic disorders, general anxiety disorders, reaction to stress, adjustment reaction, and emotional disorders. For the ADHDs, participants were considered to have an ADHD if they received one or more diagnoses of attention deficit disorder, hyperactivity disorder, hyperkinetic syndrome, or hyperkinetic conduct disorder according to the ICD-9/ICD-10 codes. The diagnosis of ADHDs in this study includes the ICD-9-CM codes “314.xx” and the ICD-10-CA codes “F90.xx” (33). All the primary diagnoses of internalizing disorders and ADHDs between 2000 (children's birth) and 2012 (children's age 10–11 years) were considered.

### Assessments of Health Behaviors

Health behaviors included diet quality, physical activity, and sedentary behavior of children aged 10–11 years. The YAQ presented detailed information on the frequency of various kinds of foods that children consume, including fruits and vegetables, grain products, meat products, and milk and alternatives (30). The YAQ also contained both macro- and micronutrients like sugar, fats, amino acids, and multivitamins and minerals (29). On the basis of the nutrient intake and dietary information of the students from the YAQ and the Canadian nutrient files (34), we calculated the intake of nutrients and the daily energy intake. We then calculated the Diet Quality Index (DQI) based on the DQI—International (DQI-I) measure (35). The DQI-I constitutes four components: variety, adequacy, moderation, and overall balance of the diet. The total DQI-I score ranges between 0 and 100, with a higher score indicating better diet quality (35). We divided the total DQI-I scores into tertiles in the analysis.

Physical activity was measured by the questions asking students and their parent/guardian(s) about: (a) travel to and from school; (b) time spent to get to and from school; (c) frequency of child's activities outside school hours; (d) activities at morning and lunch recess in the past 7 days; and (e) frequency in sports and physical activities in the 7 days. These questions, consisting of 29 items, were largely adopted from the Physical Activity Questionnaire for Children (PAQ-C) (30), which has been demonstrated to be valid and have high reliability (31). A composite score ranging from 1 to 6 was computed based on the score on each of the 29 items. For the purpose of our analysis, the PAQ-C score was dichotomized into “physically active” if a student had a score of 3 or greater and “physically inactive” if a student had a score < 3.

Sedentary behavior was measured by students' self-reported questions on the number of hours daily spent on playing computers or video games and on watching TV, with four response categories: < 1 h a day, 1–2 h a day, 3–4 h a day, and 5 h or more a day. Few students responded to the level of 5 h or more a day; hence, we categorized the SB variables into three levels: < 1 h/day, 1–2 h/day, and ≥3 h/day.

### Assessments of Health-Related Quality of Life

HRQoL was assessed by the EQ-5D-Y (youth) that was developed by the EuroQol group (https://euroqol.org/) and is suitable for use among children and youth between the ages of 8 and 18 years (32). The instrument consists of a five-dimensional descriptive system asking children whether they have (a) no problems, (b) some problems, or (c) a lot of problems on each of the following items: (i) walking; (ii) looking after myself; (iii) doing usual activities; (iv) having pain or discomfort; and (v) feeling worried, sad, or unhappy (32). The EQ-5D-Y has been previously validated and showed high reliability, and construct and discriminant validity (36). The variable for each question was dichotomized into two levels: “some problems or a lot of problems” and “no problems.”

### Assessments of Covariates

The socio-demographic characteristics of the students included child's gender, household income, highest parental educational level, and residential area. Household income was categorized into four levels: $0–$50,000, $50,001–$75,000, $75,001–$100,000, and >$100,000. Parental educational attainment was categorized into three levels: secondary school or less, college, and university or above. The residential area was classified as urban and rural based on the school location (metropolitan, city, or rural town). For body weight status, we adopted the age- and gender-specific body mass index (BMI) cutoff points for children established by the International Obesity Task Force (37) and classified body weight into normal weight, overweight, and obese. In the analysis, body weight was dichotomized as obese and non-obese (normal weight and overweight).

### Statistical Analysis

Descriptive analysis was conducted to describe the frequency distribution of students by health behaviors, the EQ-5D-Y dimensions, body weight, and socio-demographic variables. The percentage of students receiving a diagnosis of internalizing disorders and ADHDs was calculated for each group of the described variables.

To examine the associations between diet quality, PA, SB, HRQoL, and mental health disorder, we employed univariate (unadjusted) and multivariable (adjusted) negative binomial regression models (NBMs). The NBM is an appropriate method to analyze over-dispersed counts data, like the number of a diagnosis of mental health disorders by health care providers, where the variance (7.54 for internalizing disorder and 12.21 for ADHD) is greater than its mean (0.46 for internalizing disorder and 0.71 for ADHD) under the Poisson distribution. The multivariable NBM adjusted for the confounding influence of students' gender, household income, parental education, place of residency, and body weight status. The incidence rate ratio (IRR) and 95% confidence interval (CI) were reported for the regression result.

Missing values for household income (23.8%), parental education (3.1%), weight status (3.1%), and sedentary behavior (0.4% for computers/video games) were entered as separate covariate categories in the regression models, and their estimates were not presented. All analyses were weighted such that the estimates represent the population of grade five students in the province of Alberta. The STATA/IC 15 software (StataCorp. 2017 Stata Statistical Software: Release 15, StataCorp LLC, College Station, TX) was used for the statistical analysis.

### Research Ethics

The 2012 REAL Kids Alberta survey, including the parental informed consent forms, was approved by the Human Research Ethics Boards of the University of Alberta. The data linkage of the 2012 REAL Kids Alberta survey with the administrative health data was approved by the Human Research Ethics Boards of the University of Alberta and by Alberta Health and Wellness (reference no. REQ-01555).

## Results

Table 1 shows the descriptive results for the socio-demographic characteristics, health behaviors, HRQoL, and body weight status of the grade five students participating in the 2012 Alberta REAL Kids survey and the percentage of receiving a diagnosis of internalizing disorders and ADHDs from 2000 to 2012. Of the 1,352 participating students, 12.31% (*n* = 154) were diagnosed with an internalizing disorder and 8.32% (*n* = 113) were diagnosed with an ADHD during the time between birth (2000–2011) and the beginning of the survey in April in 2012 (children at age 10 or 11 years). Students who had a poorer diet quality and engaged in an inactive lifestyle received more physicians' diagnoses of internalizing disorders and ADHDs throughout childhood. Students who spent more time using a computer or playing video games (e.g., ≥3 h/day relative to < 1 h/day) had more health care provider contacts for an ADHD. The prevalence of diagnosis for an internalizing disorder was higher among students who reported having some or a lot of problems in “feeling worried, sad or unhappy” of the EQ-5D-Y, and the prevalence of diagnosis for an ADHD was greater among students who had some or a lot of problems in “having pain or discomfort.” The number of students who received a diagnosis of internalizing disorder and ADHD was higher among boys than girls.

**Table 1:** Socio-demographic characteristics, health behaviors, HRQoL, and body weight status of the grade five students participating in the 2012 REAL Kids survey, Alberta, Canada, and percentage of receiving a diagnosis of internalizing disorders and ADHDs from 2000 to 2012.

**Variables**	**% of Students** **(*n* = 1,352)**	**% of internalizing disorders**	**% of ADHDs**
Total sample	–	12.31 (*n* = 154)	8.32 (*n* = 113)
DQI			
Lowest tertile	–	12.72	9.71
Middle tertile	–	12.65	8.54
Highest tertile	–	11.57	6.76
Physical activity			
Inactive (PAQ-C score <3.0)	29.7	13.93	13.01
Active (PAQ-C score ≥3.0)	70.3	11.62	6.33
Computers/video games			
<1 h/day	40.8	12.43	4.80
1–2 h/day	31.5	13.32	7.04
≥3 h/day	27.4	11.13	14.92
Missing	0.4	–	–
TV viewing			
<1 h/day	27.4	12.78	7.87
1–2 h/day	45.7	11.88	7.07
≥3 h/day	26.9	12.68	10.98
HRQoL: the EQ-5D-Y dimensions			
Walking			
No problems	91.7	11.74	8.14
Some or a lot of problems	8.3	18.70	10.41
Looking after self			
No problems	96.4	12.37	8.09
Some or a lot of problems	3.6	11.20	14.75
Doing usual activities			
No problems	89.6	11.98	8.07
Some or a lot of problems	10.4	14.92	10.58
Having pain or discomfort			
No problems	59.0	11.35	7.66
Some or a lot of problems	41.0	13.71	9.29
Feeling worried, sad, or unhappy			
No problems	69.9	10.72	7.79
Some or a lot of problems	30.1	16.02	9.56
Obese status			
Normal weight/overweight	90.1	11.89	8.06
Obese	6.8	13.41	8.43
Missing	3.1	–	–
Gender			
Girls	52.0	10.60	4.55
Boys	48.0	13.83	12.23
Residence			
Urban	65.2	13.32	9.25
Rural	34.8	10.40	6.57
Parental education			
Secondary school or less	23.9	14.92	10.48
College	36.3	14.83	8.60
University or above	36.7	9.04	6.69
Missing	3.1	–	–
Household income (Can$)			
≤ 50,000	19.3	14.91	8.44
50,001–75,000	12.4	10.00	6.91
75,001–100,000	13.4	8.95	7.18
>100,000	31.0	13.19	7.31
Do not know/not to answer/missing	23.8	–	–

*Weighted percentages of students are presented*.

*HRQoL, health-related quality of life; ADHD, attention deficit and hyperactivity disorder; DQI, diet quality index*.

Table 2 presents the negative binomial regression results for the associations between health behaviors, and internalizing disorder and ADHD, respectively. Students in the highest tertile for diet quality had significantly lower number of diagnoses of internalizing disorder (IRR = 0.44, 95% CI = 0.23–0.85) and ADHD (IRR = 0.43, 95% CI = 0.20–0.91), respectively, relative to students in the lowest tertile for diet quality after adjusting for the effects of socio-demographic variables (adjusted model 1, Table 2). Students who were physically inactive had significantly more diagnoses of internalizing disorder (IRR = 1.98, 95% CI = 1.19–3.30) and ADHD (IRR = 4.17, 95% CI = 2.25–7.72) relative to their peers who were physically active (adjusted model 1). Children who played computers or video games more than 3 h daily had more health care contacts for ADHD than their peers who played computers or video games <1 h daily (IRR = 2.96, 95% CI = 1.37, 6.41; adjusted model 1). TV viewing was not significantly associated with increased diagnoses of internalizing disorder. Excessive TV viewing (>3 *vs*. <1 h/day) was significantly associated with increased diagnoses of ADHD in the unadjusted model (IRR = 2.43, 95% CI = 1.14–5.16), but the association was attenuated to be insignificant in the adjusted models. The adjusted associations between health behaviors and mental health disorders were similar after additional adjustment for obese status (adjusted model 2, Table 2).

**Table 2:** IRRs and 95% CIs for diagnoses of internalizing disorder and ADHD by health behaviors among grade five students participating in the 2012 REAL Kids survey, Alberta, Canada (*n* = 1,352).

**Variables**	**Internalizing disorder: IRR (95% CI)**			**ADHD: IRR (95% CI)**		
	**Unadjusted model**	**Adjusted model 1**	**Adjusted model 2**	**Unadjusted model**	**Adjusted model 1**	**Adjusted model 2**
DQI (ref.: Lowest tertile)						
Middle tertile	0.55 (0.24–1.25)	0.80 (0.43–1.51)	0.82 (0.43–1.55)	0.71 (0.34–1.48)	**0.35 (0.16–0.76)**	**0.41 (0.19–0.90)**
Highest tertile	**0.29 (0.13–0.65)**	**0.44 (0.23–0.85)**	**0.46 (0.24–0.87)**	0.57 (0.29–1.13)	**0.43 (0.20–0.91)**	**0.45 (0.21–0.97)**
Physical activity (ref.: Active)						
Inactive (PAQ-C score <3.0)	**2.31 (1.03–5.17)**	**1.98 (1.19–3.30)**	**2.01 (1.21–3.35)**	**2.43 (1.34–4.41)**	**4.17 (2.25–7.72)**	**4.36 (2.30–8.28)**
Computers/video games (ref.: <1 h/day)						
1–2 h/day	0.79 (0.39–1.60)	0.87 (0.49–1.53)	0.86 (0.49–1.52)	1.35 (0.60–3.05)	0.81 (0.39–1.68)	0.82 (0.40–1.68)
≥3 h/day	1.08 (0.35–3.32)	0.69 (0.35–1.37)	0.70 (0.36–1.39)	**3.25 (1.53–6.91)**	**2.96 (1.37–6.41)**	**3.34 (1.59–6.99)**
TV viewing (ref.: <1 h/day)						
1–2 h/day	1.33 (0.66–2.67)	1.05 (0.60–1.84)	1.03 (0.59–1.79)	1.12 (0.54–2.32)	0.92 (0.46–1.85)	0.88 (0.44–1.76)
≥3 h/day	1.65 (0.59–4.64)	1.06 (0.56–1.99)	1.05 (0.56–1.97)	**2.43 (1.14–5.16)**	1.41 (0.64–3.08)	1.31 (0.60–2.82)
Gender (ref.: Girls)						
Boys	1.10 (0.49–2.47)	**1.77 (1.04–3.00)**	**1.81 (1.07–3.05)**	**3.57 (1.76–7.21)**	**8.19 (4.56–14.72)**	**7.92 (4.41–14.22)**
Residential area (ref.: Urban)						
Rural	**0.51 (0.26–1.00)**	0.63 (0.38–1.05)	0.64 (0.39–1.06)	0.65 (0.36–1.19)	0.70 (0.40–1.24)	0.75 (0.43–1.32)
Parental education (ref.: Secondary or less)						
College	0.44 (0.18–1.08)	0.60 (0.30–1.18)	0.58 (0.29–1.15)	0.83 (0.43–1.61)	**2.03 (1.02–4.04)**	**2.03 (1.01–4.08)**
University or above	0.43 (0.16–1.15)	**0.43 (0.22–0.86)**	**0.43 (0.21–0.86)**	0.45 (0.20–1.05)	0.77 (0.38–1.56)	0.77 (0.38–1.56)
Household income (ref.: ≤ $50,000)						
$50,001–$75,000	**0.19 (0.07–0.57)**	**0.30 (0.12–0.73)**	**0.30 (0.12–0.73)**	0.74 (0.21–2.57)	1.52 (0.42–5.52)	1.48 (0.42–5.23)
$75,001–$100,000	0.52 (0.17–1.63)	0.52 (0.21–1.29)	0.53 (0.22–1.29)	0.78 (0.33–1.82)	0.53 (0.23–1.23)	0.56 (0.24–1.30)
>$100,000	0.52 (0.18–1.47)	0.78 (0.37–1.61)	0.78 (0.38–1.61)	1.00 (0.45–2.21)	1.09 (0.46–2.59)	1.11 (0.47–2.62)
Obese (yes vs. no)	0.64 (0.27–1.53)		0.87 (0.37–2.05)	**0.24 (0.09–0.62)**		**0.21 (0.08–0.53)**

*Adjusted model 1, adjusted for the demographic variables; adjusted model 2, inclusion of obese status from adjusted model 1. The adjusted models also adjusted for energy intake. All estimates were weighted to represent population estimates*.

IRRs, incidence rate ratios; CIs, confidence intervals; ADHD, attention deficit and hyperactivity disorder; ref., reference group; DQI, diet quality index. Bold values indicate statistical significance (p < 0.05).

Boys were more likely than girls to receive health care contacts for both internalizing disorder and ADHD. Obese children were less likely to be diagnosed with ADHD than normal weight children. Students with the highest level of parental education (university or above *vs*. secondary or less) experienced lower number of diagnoses of internalizing disorder (IRR = 0.43, 95% CI = 0.22–0.86), while students in a household with the highest parental education as college level (*vs*. secondary or less) had more diagnoses of ADHD (IRR = 2.03, 95% CI = 1.02–4.04) (adjusted model 1, Table 2). Children with an annual household income of $50,001–$75,000 were less likely to be diagnosed with an internalizing disorder compared to those children with the lowest annual household income (< $50,001) (Table 2).

Table 3 shows the negative binomial regression results for the association between HRQoL measured by the EQ-5D-Y and mental health disorders. Children who reported having some or a lot of problems relative to no problems in “feeling worried, sad or unhappy” had more health care provider visits for internalizing disorders (unadjusted IRR = 2.38, 95% CI = 1.07–5.29; adjusted model 1: IRR = 1.91, 95% CI = 1.05–3.47; adjusted model 2: IRR = 1.81, 95% CI = 1.06–3.07). Children who reported having some or a lot of problems in “having pain or discomfort” received more diagnoses of ADHDs based on the univariate regression result (unadjusted IRR = 1.80, 95% CI = 1.00–3.23).

**Table 3:** IRRs and 95% CIs for diagnoses of internalizing disorder and ADHD by the EQ-5D-Y dimensions among grade five students participating in the 2012 REAL Kids survey, Alberta, Canada.

**Variables**	**Internalizing disorder: IRR (95% CI)**			**ADHD: IRR (95% CI)**		
	**Unadjusted model** **(*n* = 1,352)**	**Adjusted model 1** **(*n* = 1,346)**	**Adjusted model 2** **(*n* = 1,346)**	**Unadjusted model** **(*n* = 1,352)**	**Adjusted model 1** **(*n* = 1,346)**	**Adjusted model 2** **(*n* = 1,346)**
The EQ-5D-Y dimensions (ref.: No problems)						
Walking						
Some or a lot of problems	1.25 (0.59–2.62)	1.25 (0.57–2.77)	0.96 (0.48–1.91)	1.89 (0.78–4.62)	**3.58 (1.00–12.79)**	3.01 (0.87–10.37)
Looking after self						
Some or a lot of problems	0.82 (0.24–2.79)	0.72 (0.24–2.23)	1.06 (0.35–3.26)	1.21 (0.43–3.42)	1.43 (0.45–4.52)	0.92 (0.29–2.91)
Doing usual activities						
Some or a lot of problems	0.99 (0.42–2.33)	1.45 (0.57–3.67)	1.30 (0.59–2.86)	1.11 (0.39–3.18)	0.77 (0.33–1.79)	0.67 (0.28–1.57)
Having pain or discomfort						
Some or a lot of problems	1.75 (0.79–3.85)	1.27 (0.75–2.13)	1.43 (0.87–2.34)	**1.80 (1.00–3.23)**	1.56 (0.82–2.99)	1.30 (0.67–2.52)
Feeling worried, sad, or unhappy						
Some or a lot of problems	**2.38 (1.07–5.29)**	**1.91 (1.05–3.47)**	**1.81 (1.06–3.07)**	1.46 (0.78–2.73)	1.22 (0.65–2.27)	1.34 (0.70–2.56)

*Adjusted model 1, adjusted for the variables in the table and the following demographic variables: gender, residency, parental education, and household income; adjusted model 2, additionally adjusted for health behaviors (diet quality, physical activity, use of computers/video games, and TV viewing) from the adjusted model 1. The adjusted models also adjusted for energy intake. All estimates were weighted to represent population estimates*.

*IRRs, incidence rate ratios; CIs, confidence intervals; ADHD, attention deficit and hyperactivity disorder; ref., reference group; DQI, diet quality index. Bold values indicate statistical significance (p < 0.05)*.

## Discussion

In this study, we observed that poor diet quality and low physical activity among grade five students were associated with increased diagnoses of both internalizing disorders and ADHDs during childhood. Excessive use of computer and video games and watching TV were associated with increased health care services for ADHDs throughout childhood. Students with some or a lot of problems in the EQ-5D-Y dimensions of “feeling worried, sad or unhappy” and “having pain or discomfort” had greater health care contacts with health care providers for internalizing disorders and ADHDs, respectively.

This study contributes to the existing research by investigating the associations between health behavior and HRQoL among children and their mental health disorders during childhood. Particularly, we observed that higher diet quality among children aged 10–11 years was related to less clinical diagnoses of both internalizing disorders and ADHDs during childhood. Studies among adults suggest that poor diet quality is associated with a higher risk of depression over time (38). The evidence for the relationship between overall diet quality and mental health in children and youth is inconclusive due to variabilities in the measure of diets, mental health, and the study design (13, 14). Especially, longitudinal studies that investigate the association between diet quality and mental health among children < 12 years old are still lacking (13). For example, in a systematic review that synthesized the relationship between diet and mental health in children and adolescents, there were only three prospective studies among the total of 12 included studies (17, 21, 39), and only one of the three prospective studies has analyzed the association between diet and mental health in children (39). More studies using longitudinal design in childhood are needed to reinforce the evidence. The finding in this study aligns with few prior longitudinal studies showing that poor diet quality is associated with worse internalizing mental health among adolescents (17, 40). Concerning the relationship between diet quality and externalizing problems like ADHD, the observation for the effect of diet quality on ADHD in childhood in this study is consistent with our previous research (33) and other studies showing that healthy diets are associated with less ADHDs (41, 42). Notably, the present study adds to the existing findings in the literature by presenting data on diet quality and mental disorders among a school-based cohort of preadolescent children. In this study, we used the composite measure of diet quality that was based on the DQI-I (35). One major advantage of the DQI-I is that it constitutes four relevant components, including variety, adequacy, moderation, and overall balance of the diet, and it can be used for the comparison of diet quality across countries. Previous studies have also evaluated the effect of essential nutrients on mental health, such as the association of omega-3 and omega-6 fatty acids and their balance with mental health (43). Given that food items and nutrients are usually consumed in combinations that may produce a synergistic effect on mental health, more in-depth research in the future will help to better elucidate the relationship between various forms of fatty acids and mental health disorders while accounting for the effect of other relevant nutrients (e.g., essential minerals and vitamins) (21, 44).

The health benefits of PA on mental health have been well-demonstrated in cross-sectional studies in children and adolescents (11, 12). Findings from prospective studies are less consistent (18, 45–47). We found that low PA among grade five students (relative to high PA) is related to increased diagnoses of internalizing disorders in childhood, which is in line with some longitudinal studies observing that insufficient PA is associated with more internalizing problems in children and adolescents (18, 47). We observed a significant association between higher levels of PA and fewer diagnoses of ADHD among children. This finding is in line with our previous finding in adolescents (33) and some other prospective studies revealing the health benefits of PA for ADHD (48). The findings suggest that PA may present a protective effect for ADHD.

We observed that children who engaged in excessive use of computers and video games (≥3 *vs*. <1 h/day) had significantly more diagnoses of ADHDs, which is consistent with previous studies reporting that playing computers and video games was associated with increased attention problems and ADHD among children and adolescents (33, 49, 50). While some studies observed that increased time spent on playing computers and video games was related to more internalizing disorder problems such as depression and anxiety among youth (20, 51), we did not find a significant association between the use of computers and video games and internalizing disorder in children. This observation is in line with other studies reporting that playing computers or video games was not related to poor mental health (52, 53).

The effect of TV viewing on mental health among children and adolescents is inconclusive. Some studies found a significant association between increased TV viewing and lower mental health (51, 54), and other studies did not observe a significant association (19, 55). We found that watching TV was not significantly associated with diagnoses of internalizing disorder. One possible explanation for the inconsistent findings across studies is that the association of TV viewing with mental health may be related to both the time of exposure and the content of TV viewing. Watching educational TV programs may have beneficial effects for mental health (55, 56), whereas exposure to some stress- or anxiety-stimulating contents of TV programs such as violent shows or games may exacerbate children' emotional stress and anxiety (57). Studies on the relationship between SB and mental health among children and preadolescents are still lacking as the majority of the prior relevant studies in young people have examined the relationship among adolescents and youth. More research is needed to examine the relationship between different types of sedentary behaviors and mental health among children.

To the best of our knowledge, this is the first study that examined the correlations between HRQoL and mental health among a population-based sample of children using the EQ-5D-Y. Previous studies that used the EQ-5D-Y have mostly investigated the psychometric properties (e.g., validity and reliability) of the measure or applied the measure among children and youth with chronic diseases (36, 58). In this study, we found that “feeling more worried, sad or unhappy” is associated with a higher rate of internalizing disorder diagnosis and that “having more pain or discomfort” is related to a higher rate of ADHD diagnosis, which are in agreement with several other studies presenting that a lower HRQoL (both the physical and mental dimensions) is associated with both more internalizing and externalizing problems among children and adolescents (4, 27, 59). We observed in this study that 41% of children reported having “some or a lot of problems” in the EQ-5D-Y dimension “pain or discomfort” (Table 1). This finding is consistent with our previous studies that showed a similar pattern in the prevalence of health problems in the EQ-5D-Y dimensions (60).

In this study, we performed retrospective data analysis and focused on the association between health behaviors and HRQoL (exposures), respectively, among grade five students and mental health disorder outcomes throughout childhood (as lifetime diagnoses of internalizing disorder and ADHD). Prior research has shown that health behaviors and dietary habits are usually established during childhood and remain later on in adolescence or adulthood (22, 23). Our findings emphasize the importance of interventions targeting promoting healthy lifestyle behaviors to improve mental health in childhood and preadolescence. It is worth mentioning that bidirectional or reverse associations between health behaviors or HRQoL and mental health may exist. For example, health-related behaviors or HRQoL and mental health may have reciprocal associations over time (61). Longitudinal data with mental health, health behaviors, and HRQoL data available over time would warrant better elucidation of the longitudinal reciprocal associations between these variables. In this study, while the mental health disorder data were available from children's birth to the time when they were 10 or 11 years old, as students' health behaviors and HRQoL were collected at the ages of 10–11 years, and no earlier data on these variables were available, the study is limited to testing whether mental health disorders were influenced by earlier health behaviors or HRQoL among children before the ages of 10–11 years.

The observation that boys were more likely than girls to seek health care for internalizing disorder and ADHD is consistent with our previous studies in cohorts of adolescents (15, 20, 33). While some previous studies have shown that adolescent girls had more internalizing disorder problems than adolescent boys, this study observed that boys appeared more vulnerable to internalizing disorders occurring in childhood than girls. The difference may be explained by differences in the developmental trajectories of mental health problems by gender and age of children. Previous research showed that internalizing disorders such as depression, emotional problems, and anxiety increase markedly in the adolescent period, and the increase tends to be greater among girls than boys after 10–11 years (2, 62). During childhood, boys may be more subject to mental health problems than girls (62, 63). More research among children would warrant confirming the observation of gender differences in internalizing disorder problems. The findings of the present study suggest that programs targeting preventions of internalizing disorders and ADHD in childhood should be gender-focused and emphasize health promotion efforts to meet the needs among boys in order to reduce the burden of mental health disorders.

This study has several strengths. The analysis was based on a large sample of grade five students in the Canadian province of Alberta with their lifetime administrative health records linked to the population health survey data. The availability of the lifetime longitudinal administrative health data for children provided a unique opportunity for us to examine the relationship between the health behaviors and HRQoL of children and the clinical diagnoses of both internalizing disorders and ADHDs throughout childhood. The use of physician-diagnosed internalizing disorders and ADHD yielded a more accurate and clinically meaningful assessment of mental health relative to self-reported mental health measures among children. The regression analysis for the associations of mental health disorder with health behaviors and HRQoL adjusted for the confounding influence of socio-demographic variables and childhood obesity among children; thus, the findings provide more valid and robust inferences to the target population.

Limitations of this study should also be acknowledged. Assessments of diet quality, SB, and PA were based on self-report and therefore may be subject to recall bias or error. Due to the observational nature of this study, the study findings preclude causal inferences, although retrospective medical care data are better than cross-sectional studies to elucidate directionality between the predictor variables and disease outcomes. In addition, ~41% of the students who completed the REAL Kids Alberta survey were not included in the analysis because their parents did not provide valid health care card numbers for their children. However, there was not a significant difference between the participating and the non-participating students with respect to the demographic characteristics and the exposure variables, except that the prevalence of “having pain or discomfort” in the HRQoL was slightly lower in the study sample (41.22%) than in the excluded sample (45.68%, *p* = 0.032).

## Conclusions

This study revealed that low diet quality, physical inactivity, high sedentary behavior, and poor HRQoL among children were associated with more diagnoses of mental health disorders throughout childhood. Taking into consideration the growing body of literature suggesting a role of health behaviors in the causation of mental health, our findings suggest that effective health promotion programs targeting promoting healthy diet quality and physical activity, reducing sedentary behavior, and improving HRQoL among children may contribute to reducing the burden of mental health.

## Data Availability Statement

The datasets presented in this article are not readily available due to privacy and ethical restrictions. Details of the administrative health data access process are available from: https://www.alberta.ca/health-research.aspx. Requests to access the datasets should be directed to PV, paulus.veugelers@ualberta.ca.

## Ethics Statement

The 2012 RealKids Alberta survey data collection, including parental informed consent forms, were approved by the Human Research Ethics Boards of the University of Alberta. The data linkage of the 2012 RealKids Alberta survey with the administrative health data was approved by the Human Research Ethics Boards of the University of Alberta and by Alberta Health and Wellness (Reference number: REQ-01555). Written informed consent to participate in this study was provided by the participants' legal guardian/next of kin.

## Author Contributions

PV and AO conceived and designed the study. XW analyzed the data. PV, AO, and XW wrote the manuscript. All authors contributed to the article and approved the submitted version.

## Conflict of Interest

The authors declare that the research was conducted in the absence of any commercial or financial relationships that could be construed as a potential conflict of interest.
